# Sheep Are Susceptible to the Bovine Adapted Transmissible Mink Encephalopathy Agent by Intracranial Inoculation and Have Evidence of Infectivity in Lymphoid Tissues

**DOI:** 10.3389/fvets.2019.00430

**Published:** 2019-11-29

**Authors:** Eric D. Cassmann, S. Jo Moore, Jodi D. Smith, Justin J. Greenlee

**Affiliations:** ^1^Virus and Prion Research Unit, United States Department of Agriculture, National Animal Disease Center, Agricultural Research Service, Ames, IA, United States; ^2^Department of Veterinary Pathology, Iowa State University, Ames, IA, United States

**Keywords:** prion diseases, prion protein, *PRNP*, PrP^Sc^, sheep, transmissible mink encephalopathy, transmissible spongiform encephalopathies

## Abstract

Transmissible mink encephalopathy (TME) is a food borne prion disease. Epidemiological and experimental evidence suggests similarities between the agents of TME and L-BSE. This experiment demonstrates the susceptibility of four different genotypes of sheep to the bovine adapted TME agent by intracranial inoculation. The four genotypes of sheep used in this experiment had polymorphisms corresponding to codons 136, 154, and 171 of the prion gene: V_136_R_154_Q_171_/VRQ, VRQ/ARQ, ARQ/ARQ, and ARQ/ARR. All intracranially inoculated sheep without comorbidities (15/15) developed clinical signs and had detectable PrP^Sc^ by immunohistochemistry, western blot, and enzyme immunoassay (EIA). The mean incubation periods in sheep with bovine adapted TME correlated with their relative genotypic susceptibility. There was peripheral distribution of PrP^Sc^ in the trigeminal ganglion and neuromuscular spindles; however, unlike classical scrapie and C-BSE in sheep, sheep inoculated with the bovine TME agent did not have immunohistochemically detectable PrP^Sc^ in the lymphoid tissue. To rule out the presence of infectivity, the lymph nodes of two sheep genotypes, VRQ/VRQ, and ARQ/ARQ, were bioassayed in transgenic mice expressing ovine prion protein. Mice intracranially inoculated with retropharyngeal lymph node from a VRQ/VRQ sheep were EIA positive (3/17) indicating that sheep inoculated with the bovine TME agent harbor infectivity in their lymph nodes despite a lack of detection with conventional immunoassays. Western blot analysis demonstrated similarities in the migration patterns between bovine TME in sheep, the bovine adapted TME inoculum, and L-BSE. Overall, these results demonstrate that sheep are susceptible to the bovine adapted TME agent, and the tissue distribution of PrP^Sc^ in sheep with bovine TME is distinct from classical scrapie.

## Introduction

Transmissible spongiform encephalopathies (TSE) are infectious fatal neurodegenerative diseases caused by a misfolded form of the prion protein (PrP^Sc^) (1, 2). There are numerous naturally occurring prion diseases in animals including bovine spongiform encephalopathy (BSE), scrapie in small ruminants, chronic wasting disease (CWD) in cervids, transmissible mink encephalopathy (TME), Creutzfeldt-Jakob disease (CJD) in humans, and camel prion disease (3–6). Genetically susceptible animals develop disease spontaneously or after exposure to contaminated feedstuffs, environments, or infected animals.

Despite the infectious nature of PrP^Sc^, there is often a hardy species barrier that precludes cross-species transmission (7). Nevertheless, food-borne prion diseases may infect humans and have economically devastating consequences on trade. Beginning in the mid-1980's and peaking in 1996, the UK experienced an epizootic of BSE (classical BSE or C-BSE) that evidenced the zoonotic potential of prion diseases (8). A variant of Creutzfeldt-Jakob disease (vCJD) was identified in a subset of humans exposed to the agent of C-BSE in contaminated beef products (9–12). Due to the fatal consequences associated with zoonotic prions within the human food supply, substantial investigation has aimed at determining the origin of C-BSE. While the etiology of the original C-BSE case remains uncertain, it has been determined that feedback of BSE infected bovine carcasses likely amplified the outbreak (8, 13).

In addition to C-BSE, atypical forms of BSE (L-type and H-type) occur spontaneously in older cattle at a much lower frequency (4). Atypical BSE types have distinct molecular phenotypes relative to C-BSE that are demonstrable by a lower or higher molecular mass of the unglycosylated PrP^Sc^ glycoform (14). One standing theory for the origin of C-BSE is its emergence from atypical BSE and subsequent transmission between cattle. This is supported by the emergence of classical BSE phenotypes after serial transmission of H-BSE strains in wild type mice (15, 16).

Like C-BSE, transmissible mink encephalopathy (TME) is considered to be a food-borne TSE. In a 1985 outbreak of TME in Stetsonville, WI, a TSE-affected downer cow may have been the source of prion disease in infected mink. Subsequent investigations revealed a lack of strain de-adaptation after transmission of TME between cattle and mink, reinforcing the hypothesis of a common origin (5). Furthermore, bovine passaged TME (bTME) is phenotypically similar to L-BSE suggesting that a downer cow may have been the source of TSE to the mink (17). This scenario supports the occurrence of interspecies transmission between livestock in an agricultural setting. With the aim of further characterizing the host range of the TME agent, we investigated the susceptibility and disease phenotype of bovine adapted TME in sheep. Since bTME has previously been demonstrated as similar to L-BSE, we also discuss the similarities between ovine passaged bTME (o-bTME) and previously published ovine L-BSE.

## Materials and Methods

### Animal Procedures

Experimental animals were sourced from a known scrapie-free flock housed at the USDA National Animal Disease Center in Ames, IA. Approval from the Institutional Animal Care and Use Committee was procured prior to conducting this experiment (protocol number ARS-2871). Prion protein (*PRNP*) genotypes were sequenced using previously described methods (18). Amino acid sequences of the prion protein were predicted from the genotypes; they were determined to be homozygous at other known polymorphic sites G127, M137, S138, L141, R151, R154, M157, N176, H180, Q189, T195, T196, R211, Q220, and R223. Two sheep in the intracranial inoculation group were heterozygous MT112. Experimental groups were composed of sheep with polymorphisms at codons 136 and 171; there were four genotypes included in this experiment: V_136_R_154_Q_171_/VRQ, VRQ/ARQ, ARQ/ARQ, and ARQ/ARR.

### Inoculum Preparation

In order to assess the significance of bovine passaged TME on the pathobiological outcomes in sheep, a bovine adapted TME isolate was used for this experiment. In a previous experiment, the agent of transmissible mink encephalopathy was passaged two times in cattle via intracranial inoculation (19). The inoculum for this experiment was passage a third time and then prepared from brainstem at the level of the obex. The final inoculum was prepared as a 10% (w/v) homogenate.

### Animal Inoculation

Experimental inoculation of sheep was performed by two routes: oronasal and intracranial. The number of sheep for each genotype inoculated intracranially was VRQ/VRQ, *n* = 5; VRQ/ARQ, *n* = 4; ARQ/ARQ, *n* = 4; and ARQ/ARR, *n* = 4. The procedure for intracranial inoculation was performed as previously described (20–22) using 1 ml of brain homogenate. Oronasal inoculation also was performed as previously described and demonstrated to be effective for the transmission of the scrapie agent (23, 24). Briefly, the head was elevated slightly, and 1 ml of the inoculum was deposited into the right nostril via a needle-less syringe. The number of sheep inoculated oronasally for each genotype was VRQ/VRQ, *n* = 4; VRQ/ARQ, *n* = 4; and ARQ/ARQ, *n* = 4. Oronasally inoculated sheep were 3 months old at the time of inoculation.

Following inoculation, all sheep were housed for 2 weeks in a biosafety level 2 facility. Subsequently, sheep were housed outside in pens at the National Animal Disease Center where they were fed a pelleted growth and alfalfa hay ration.

The sheep were examined for signs of clinical scrapie on a daily basis. When unequivocal neurologic signs were noted, spontaneous intercurrent death occurred, or the experimental endpoint was reached, necropsy was performed on each animal.

### Sample Collection

The procedure for collection of samples was performed similar to other experiments (22). At necropsy, duplicate tissue samples were collected and stored in 10% buffered neutral formalin (globes in Bouin's fixative) or frozen. Tissues collected included brain, spinal cord, pituitary, trigeminal ganglia, eyes, sciatic nerve, third eyelid, palatine tonsil, pharyngeal tonsil, lymph nodes (mesenteric, retropharyngeal, prescapular, and popliteal), spleen, esophagus, forestomaches, intestines, rectal mucosa, thymus, liver, kidney, urinary bladder, pancreas, salivary gland, thyroid gland, adrenal gland, trachea, lung, turbinate, nasal planum, heart, tongue, and muscles (masseter, diaphragm, triceps brachii, biceps femoris, and psoas major).

### Microscopic and Immunohistochemical Evaluation

For the evaluation of spongiform change, hematoxylin and eosin stained sections of cerebrum, brainstem and cerebellum from corresponding levels at the forebrain, basal nuclei, thalamus, midbrain, pons, medulla oblongata, and obex were assessed by brightfield microscopy and scored as positive or negative.

To study the accumulation of PrP^Sc^ in tissue, brightfield microscopy was used in combination with immunohistochemistry (IHC) similar to previously described methods (20–22, 25). Briefly, paraffin-embedded tissues were sectioned at their optimum thickness (brain 4 μm, lymphoid 3 μm, other 5 μm). The presence of PrP^Sc^ and the pathologic phenotype (immunolabeling pattern and distribution) were evaluated by application of a cocktail containing two monoclonal antibodies (F89/160.1.5 and F99/97.6.1) each used at a concentration of 5 μg/ml utilizing an automated processor (Ventana Nexes, Ventana Medical Systems, Inc., Tucson, AZ). Immunohistochemical labeling patterns for PrP^Sc^ were evaluated. Explanations of the immunolabeling patterns of PrP^Sc^ have been published previously in detail (26–28).

### Western Blot

We evaluated the molecular characteristics of PrP^Sc^ using western blot detection on frozen sections of brain. Representative samples of each genotype and inoculation group were assessed and compared to selected TSEs of interest. Acetone precipitation was used for the blot comparing multiple TSE strains. The tissue volume equivalent (mg eq) loaded for scrapie, o-bTME, bTME, and L-BSE was 0.3, 1.5, 1.5, and 1.5 mg, respectively. Acetone precipitation was performed as follows. Proteinase K treated brain homogenates were mixed with four volumes of −20°C acetone and incubated 1.5 h at −20°C. The samples were pelleted (15,000 rcf for 10 min at 10°C). The supernatant was discarded, and the pellet was resuspended in 1× LDS loading buffer containing 1 μL of β-mercaptoethanol. Protein gel electrophoresis and subsequent immunoblotting procedures were performed as previously described (20) utilizing murine monoclonal antibodies Sha31 (diluted to a final concentration of 0.15 μg/ml; Cayman Chemical Company, Ann Arbor, MI) or P4 (diluted to a final concentration of 0.1 μg/ml; R-Biopharm, Inc., Marshall, MI), a biotinylated anti-mouse secondary antibody (diluted to a final concentration of 0.1 μg/ml; Biotinylated anti-mouse IgG, Amersham Biosciences, USA), and streptavidin conjugated to horseradish-peroxidase (diluted to a final concentration of 0.1 μg/ml; Streptavidin horseradish-peroxidase conjugate, Amersham Biosciences, USA). For detection and image acquisition, a chemiluminescent and chemifluorescent (HRP) substrate (Pierce ECL-Plus, ThermoFisher Scientific, Waltham, MA) was used in conjunction with a gel imaging system (G:BOX Chemi-XT4, Syngene, Frederick, MD). Analysis of glycoform profiles was carried out with Image Lab^TM^ (version 6.0.1) software (Bio-Rad Laboratories, Hercules, CA).

### Enzyme Immunoassay

A commercially available enzyme immunoassay (HerdChek^®^, IDEXX Laboratories Inc., Westbrook, ME) was used to screen for the presence of PrP^Sc^ in brainstem at the level of the obex and the retropharyngeal lymph node (RPLN). Assays were conducted according to kit instructions except that the samples were prepared as a 20% (w/v) tissue homogenate. Cut-off numbers were determined with a negative control per kit instructions; values greater than the mean optical density (O.D.) of negative controls +0.180 were considered positive for the purposes of screening samples.

### Transgenic Mouse Bioassays

In order to determine if there was infectivity in the IHC negative lymph nodes, we performed a bioassay with transgenic mice expressing ovine *PRNP* (Tg338) (29). The RPLN of sheep with VRQ/VRQ or ARQ/ARQ genotypes were used as inocula (sheep 935 and 808, respectively). Tg338 mice were inoculated intraperitoneally with 100 μl of 1% w/v RPLN homogenate from VRQ/VRQ (*n* = 18) or ARQ/ARQ (*n* = 14) sheep. Separate groups of mice were intracranially inoculated with VRQ/VRQ (*n* = 17) or ARQ/ARQ (*n* = 18) RPLN inocula. Intracranial and intraperitoneal inoculation routes were used separately to assess potential differences of inoculation route on the transmission efficiency. Mice were monitored daily for the appearance of clinical signs. At the time of death or at the end of study, brains were tested for PrP^Sc^ by EIA. The attack rate was defined as the percentage of mice from an experimental group that had a positive EIA result. Incubation periods were expressed as the mean number of days post-inoculation for mice with positive EIA results. To determine the attack rate, mice dying from intercurrent disease or without neurologic signs that survived 2 SD less than the mean incubation period of positive mice were excluded.

### Survival Analyses

Survival analyses for all experiments were performed using Graphpad Prism 7 (GraphPad Software, San Diego, CA). Animals were censored only if they were negative and died from intercurrent disease before the experimental end-point.

### Definitions

To analyze experimental data, the following definitions were utilized in agreement with previous reports (22). In the sheep experiment, the attack rate is defined as the percentage of animals from an experimental group that had detectable PrP^Sc^ in any tissue. Incubation period is defined as the amount of time that elapsed between inoculation and euthanasia at the onset of clinical signs. The agent of TME passaged in cattle is referred to in the text bovine TME (bTME); whereas, ovine passaged bTME is abbreviated as o-bTME.

## Results

### Sheep Were Susceptible to the Bovine Adapted TME Agent by Intracranial Inoculation

**Figure 1:**
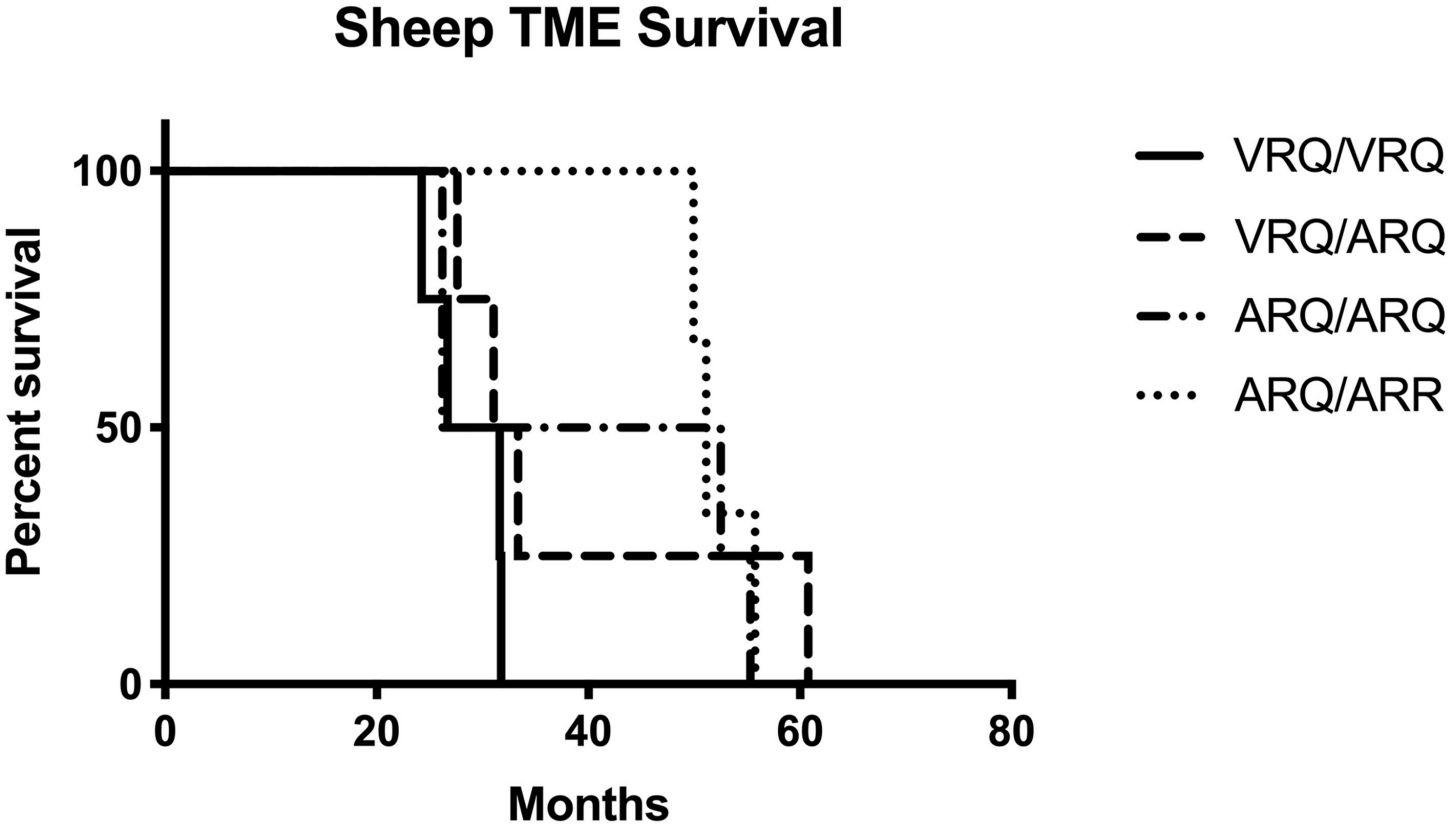
Survival plot of sheep inoculated intracranially with the agent of TME. The shortest incubation period was observed in sheep with the VRQ/VRQ genotype indicated by a solid black line.

In order to test the susceptibility of sheep to the bTME agent, we intracranially inoculated 17 sheep that had four different *PRNP* polymorphisms. All experimental sheep in the intracranial inoculation group developed scrapie-like neurologic disease except for two sheep. These two sheep were culled due to intercurrent disease at 10 months post-inoculation. Neither of the culled sheep had detectable PrP^Sc^ in the CNS or peripheral tissues. Excluding animals with intercurrent disease that were removed from the study early, the attack rate was 100% (15/15) and the mean incubation periods were 29, 38, 40, and 52 months for VRQ/VRQ, VRQ/ARQ, ARQ/ARQ, and ARQ/ARR genotypes, respectively. A survival plot for the intracranial inoculation group is illustrated in Figure 1.

### Intracranially Inoculated Sheep Developed Spongiform Encephalopathy and Had Widespread PrP^**Sc**^ Accumulation in the Brain

Hematoxylin and eosin stained sections of brain were evaluated for spongiform change in each animal. All genotypes of intracranially inoculated sheep had diffuse spongiform encephalopathy except for two sheep that were euthanized at 10 months post-inoculation due to intercurrent disease (Table 1).

**Table 1:** Experimental findings in sheep intracranially inoculated with the TME agent.

			**Immunohistochemistry (PrP^Sc^)**								**Brainstem**	
**Ear Tag**	**Genotype**	**Incubation**	**Brainstem**	**Trigeminal**	**PHT**	**PAL**	**RPLN**	**RAMALT**	**Peripheral**	**ENS**	**SE**	**EIA**
	***PRNP***	**Period (MPI)**		**Ganglia**					**Muscle NMS**			
962	VRQ/VRQ	32	+	+	–	–	–	–	M	–	+	+
935	VRQ/VRQ	27	+	+	–	–	–	–	P	–	+	+
811	VRQ/VRQ	32	+	+	–	–	–	–	M	–	+	+
945	VRQ/VRQ	10^a^	–	NA	–	–	–	–	–	–	–	–
957	VRQ/VRQ	24	+	+	–	–	–	–	–	–	+	NA
816	VRQ/ARQ	28	+	+	–	–	–	–	–	–	+	+
818	VRQ/ARQ	61	+	-	–	–	–	–	–	–	+	+
834	VRQ/ARQ	33	+	+	–	–	–	–	M	–	+	+
841	VRQ/ARQ	31	+	NA	–	–	–	–	-	–	+	+
808	ARQ/ARQ	26	+	NA	–	–	–	NA	NA	–	+	+
815	ARQ/ARQ	53	+	+	–	–	–	–	–	–	+	+
835	ARQ/ARQ	55	+	+	–	–	–	–	P	+	+	+
936	ARQ/ARQ	26	+	NA	–	–	–	–	–	–	+	+
833	ARQ/ARR	50	+	–	–	–	–	–	–	–	+	+
951	ARQ/ARR	51	+	–	–	–	–	–	–	–	+	+
955	ARQ/ARR	10^a^	–	–	–	–	–	–	–	–	-	-
970	ARQ/ARR	56	+	–	–	–	–	–	–	–	+	+

*PNRP, prion protein gene; MPI, months post-inoculation; PHT, pharyngeal tonsil; PAL, palatine tonsil; RPLN, retropharyngeal lymph node; RAMALT, Recto-anal mucosa associated lymphoid tissue; NMS, neuromuscular spindle; ENS, enteric nervous system; SE, spongiform encephalopathy; EIA, enzyme immunoassay; IC, intracranial; NA, not available; M, masseter; P, psoas major*.

a *Culled or died from intercurrent disease*.

**Figure 2:**
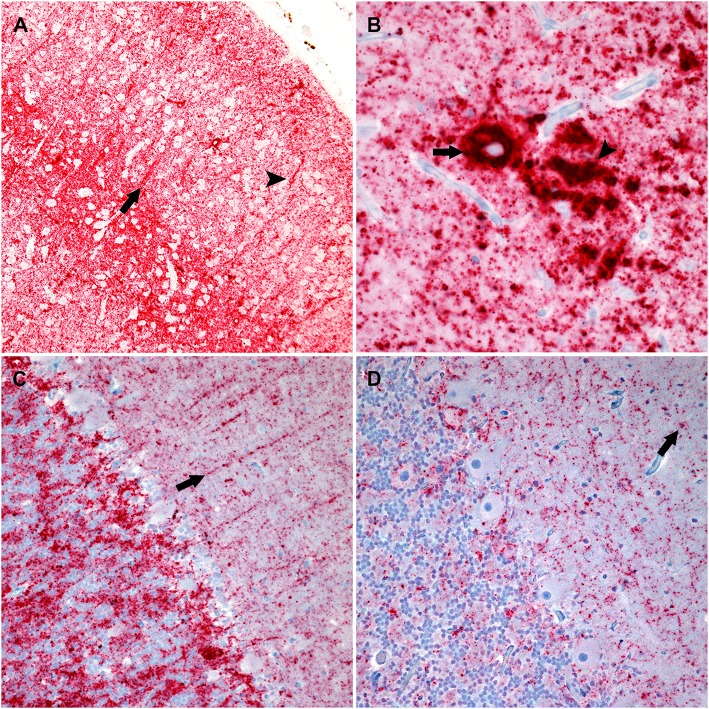
Photomicrographs of brain regions from sheep that were inoculated intracranially with the agent of bTME. Immunohistochemistry for PrP^Sc^, monoclonal antibodies F89/160.1.5, and F99/97.6.1. **(A)** PrP^Sc^ in the frontal neocortex from a sheep with an ARQ/ARR genotype. In this genotype there is diffuse particulate that is denser in neocortical layers IV and V. There also are prominent linear (arrow) and perineuronal (arrowhead) immunolabeling patterns. **(B)** In the cerebrum from sheep with the ARQ/ARR genotype, there are vascular plaque-like accumulations (arrow) of PrP^Sc^. The arrowhead indicates an area of coalescing particulate immunolabeling. **(C)** Cerebellum from a sheep with an VRQ/ARQ genotype. In the granular layer there is particulate and intraglial immunolabeling, and in this image, linear type PrP^Sc^ (arrow) is evident in the molecular layer. The immunolabeling types present are typical of the VRQ/VRQ, VRQ/ARQ, and ARQ/ARQ genotypes. **(D)** Cerebellum from a sheep with the ARQ/ARR genotype. Overall, there is less PrP^Sc^ immunolabeling. In the selected image, there is primarily diffuse particulate with intermittent intraglial (arrow) PrP^Sc^.

A cocktail of two antibodies, F89/160.1.5 and F99/97.6.1, directed against PrP^Sc^ was used to assess the distribution and immunolabeling types of PrP^Sc^ in the brain. There were similar PrP^Sc^ immunolabeling profiles in the cerebrum and brainstem of each genotype characterized by particulate, intraglial, stellate, perineuronal, and linear types. The sheep with an ARQ/ARR genotype varied by having more prominent linear and perineuronal labeling types in the neocortex (Figure 2A). This genotype also contained areas with vascular plaque-like accumulations and coalescing PrP^Sc^ (Figure 2B).

The cerebellar PrP^Sc^ immunolabeling patterns were similar between all genotypes except ARQ/ARR with gray matter particulate and intraglial labeling in the granule cell layer accompanied by particulate, perineuronal and linear in the molecular layer (Figure 2C). There was minimal white matter PrP^Sc^ labeling in the cerebellum. The ARQ/ARR genotype had overall less immunolabeling; however, when present, there were patchy areas of intraglial, perineuronal, intraneuronal, and particulate labeling patterns (Figure 2D).

The cervical, thoracic, and lumbar spinal cords contained PrP^Sc^ in all genotypes. There was diffuse accumulation of PrP^Sc^ in the gray matter that was concentrated primarily in the dorsal horn. Immunolabeling types included particulate, intraglial, intraneuronal, and sometimes perineuronal.

### Immunoreactivity Was Present in the Peripheral Nervous System but Not Lymphoid Tissues

**Figure 3:**
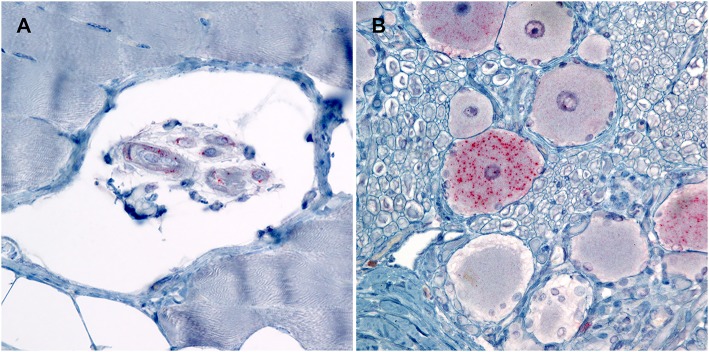
Photomicrographs of PrP^Sc^ in peripheral tissues from sheep that were inoculated intracranially with the agent of TME. Immunohistochemistry for PrP^Sc^, monoclonal antibodies F89/160.1.5 and F99/97.6.1. **(A)** There is immunolabeling of PrP^Sc^ within the neuromuscular spindles of the masseter muscle of sheep #962 (VRQ/VRQ). **(B)** There is intraneuronal immunolabeling of PrP^Sc^ in the trigeminal ganglion of sheep #834 (VRQ/ARQ).

In order to evaluate peripheral tissue for the presence of PrP^Sc^, we applied PrP^Sc^ specific antibodies, F89/160.1.5 and F99/97.6.1, to formalin fixed paraffin embedded tissue sections. We detected PrP^Sc^ in peripheral tissues of all four genotypes (Table 2) of sheep. Various sheep of the VRQ/VRQ, VRQ/ARQ, and ARQ/ARQ genotypes had PrP^Sc^ within the neuromuscular spindles of the masseter (Figure 3A) and psoas major muscles. Except for the ARQ/ARR genotype, most sheep also had detectable immunolabeling of PrP^Sc^ in the trigeminal ganglia (Figure 3B). Accumulation of PrP^Sc^ in the trigeminal ganglia and neuromuscular spindles is similar to the distribution of L-BSE in sheep (30). All genotypes of sheep had retinal PrP^Sc^ accumulation. A single sheep from the ARQ/ARR genotype group had PrP^Sc^ labeling in the myenteric plexus of the small intestinal enteric nervous system. Other tissues had variable PrP^Sc^ accumulation including the sciatic nerve, adrenal medulla and pituitary gland. Unlike classical scrapie and C-BSE in sheep, there was no immunohistochemical detection of PrP^Sc^ in lymphoreticular tissue.

**Table 2:** Immunolabeling of PrP^Sc^ in tissue organized by genotype of intracranially inoculated Suffolk sheep.

	**VRQ/VRQ**	**VRQ/ARQ**	**ARQ/ARQ**	**ARQ/ARR**
	**# Positive/*n***	**# Positive/*n***	**# Positive/*n***	**# Positive/*n***
Total number of sheep	4/5^a^	4/4	4/4	3/4^a^
Excluding intercurrent disease	4/4	4/4	4/4	3/3
Mean IP of positive sheep (months)	29	38	40	52
**Tissue**				
**Muscle**				
Masseter	+	+	0	0
Psoas major	+	0	+	0
**Nervous: Non-CNS**				
Retina	+	+	+	+
Trigeminal ganglia	+	+	+	0
Sciatic nerve	+	0	0	0
Adrenal medulla	+	+	0	0
Myenteric plexus (SI)	0	0	+	0
**CNS**				
Cerebrum	+	+	+	+
Cerebellum	+	+	+	+
Brainstem	+	+	+	+
Spinal cord	+	+	+	+
**Lymphoreticular**	0	0	0	0
**Other**				
Pituitary gland	+	+	+	+

a *Sheep culled or died from intercurrent disease*.

*IP, incubation period; SI, small Intestine; +, Positive; 0, Negative*.

### Orally Inoculated Sheep Lacked Spongiform Change and Did Not Have Immunoreactivity for PrP^Sc^

We assessed the susceptibility of sheep to bTME agent after oronasal exposure. Two sheep were culled 9–11 months early due to recurring lameness. After an observation period of 58–69 months, none of the oronasally inoculated animals (0/12) had spongiform encephalopathy nor detectable PrP^Sc^ by IHC in brainstem at the level of the obex. The RPLN, palatine tonsil, pharyngeal tonsil, and small intestines were also negative for PrP^Sc^ by IHC. Misfolded prion proteins were not detected in the brainstems nor RPLNs by enzyme immunoassay (EIA).

### The Molecular Profiles of Brain Tissues From Sheep or Cattle With TME Can Be Distinguished by Western Blot Using P4 Antibody

**Figure 4:**
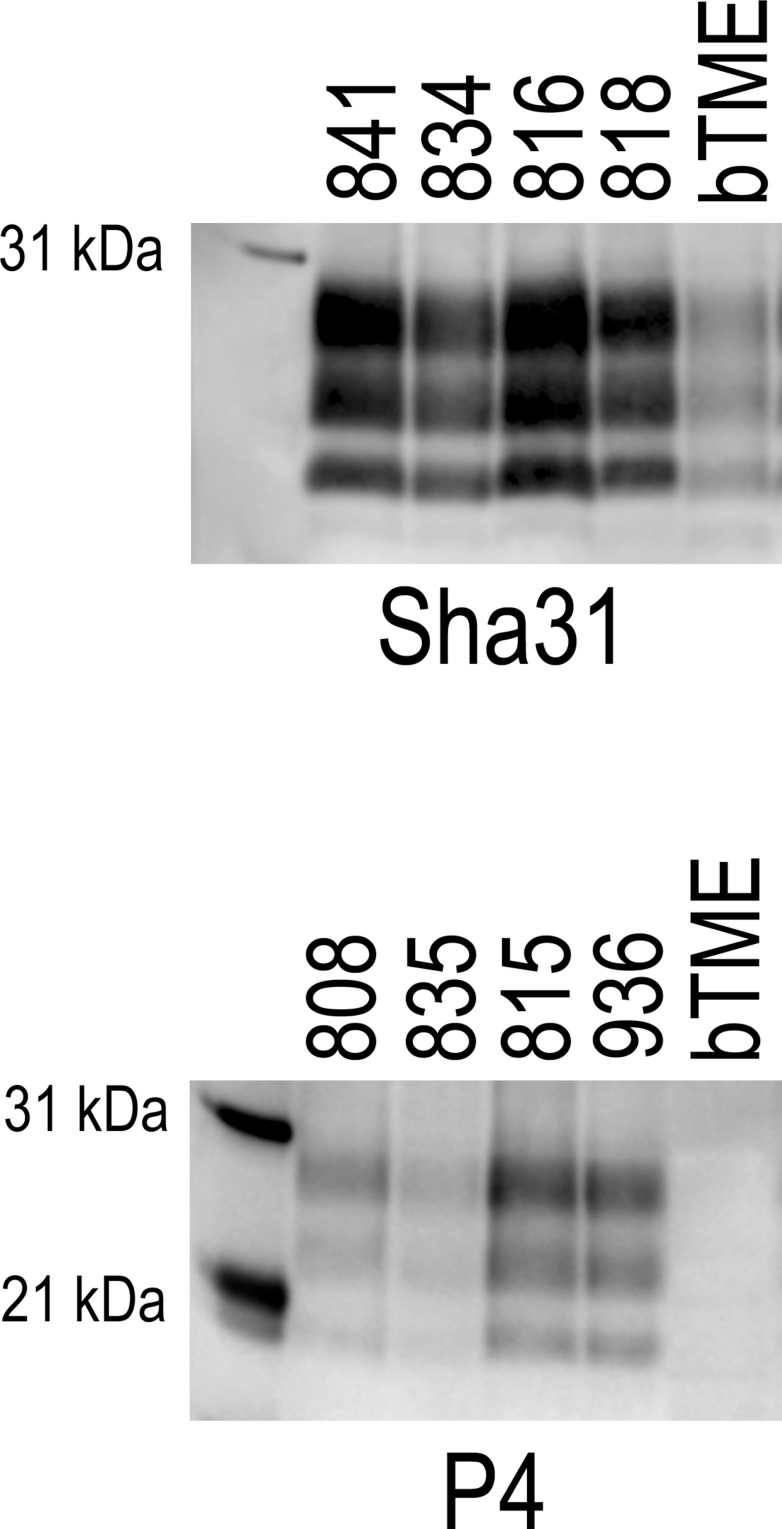
Western blots of brain samples from sheep that were inoculated intracranially with the bovine passaged TME agent (bTME). The blots were immunolabeled with either the Sha31 (top) or P4 (bottom) antibody. **Sha31**, The antibody Sha31 was used on brain homogenates from sheep with the VRQ/ARQ genotype. The banding patterns are similar to the inoculum (bTME). **P4**, The antibody P4 is immunoreactive with brain homogenates from sheep with the ARQ/ARQ genotype. The inoculum, bTME, does not bind with the P4 antibody.

We were able to discriminate o-bTME from bTME. We analyzed the banding patterns o-bTME using two separate antibodies that can discriminate scrapie from C-BSE and L-BSE (31–33). Both o-bTME and bTME were immunoreactive for Sha31 (Figure 4). With the P4 antibody, o-bTME was immunoreactive; however, bTME was not immunoreactive.

**Figure 5:**
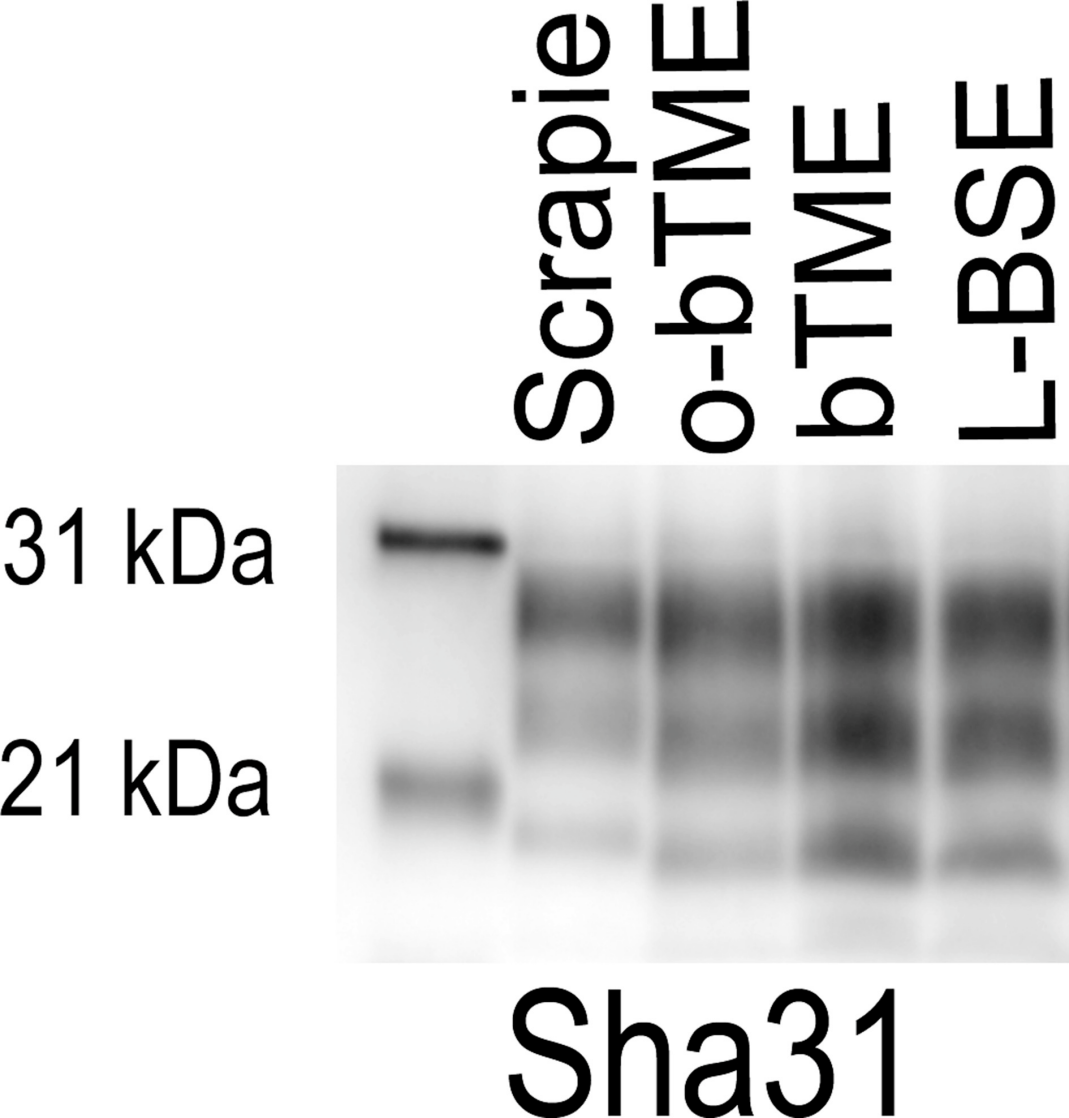
Western blot comparing multiple ruminant TSEs including L-BSE, scrapie, bovine TME (bTME), and ovine passaged bTME (o-bTME). The blot was immunolabeled with Sha31 anti-PrP antibody after acetone precipitation. The characteristics of bTME in sheep are similar to 3rd passage bTME in cattle that resembles L-BSE. Scrapie has a distinct migration pattern.

**Figure 6:**
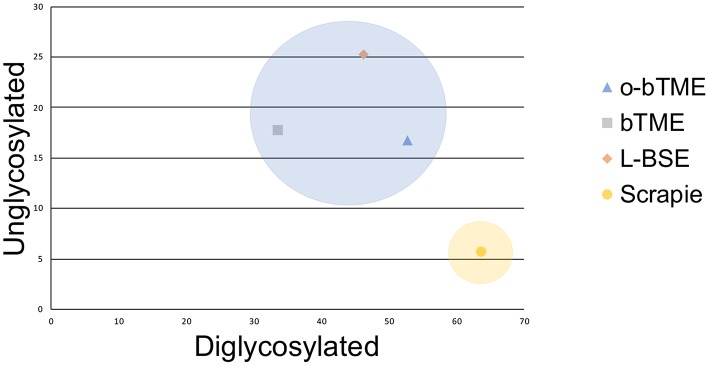
Plotted unglycosylated and diglycosylated PrP^Sc^ as a percentage of total PrP^Sc^ residue in different ruminant TSEs. The passaged TME isolates and L-BSE are more similar and cluster closer together than sheep scrapie.

In order to further characterize the molecular profile of o-bTME, we compared it to other relevant prion strains of sheep and cattle using the Sha31 antibody (Figure 5). Different TSE strains vary by their molecular banding pattern (molecular weight) of the diglycosylated, monoglycosylated, and unglycosylated PrP^Sc^ fragments (34). All samples for this blot were acetone precipitated, and the sample amounts were adjusted to obtain optimal banding densities. Overall, the banding pattern of o-bTME resembled the inoculum, third passage bTME (also seen in Figure 4 using non-enriched samples). The molecular profile of L-BSE was similar to both ovine and cattle passaged TME isolates. Scrapie had a distinct banding pattern compared to the TME and L-BSE samples (Figure 6). We did not observe intergenotypic differences in the molecular properties of o-bTME.

### Enzyme Immunoassays for Misfolded Proteins Were Positive in Samples From the Brains of Intracranially Inoculated Sheep

As a rapid screening method for PrP^Sc^, we used EIA to test the brainstem and lymph nodes of all experimental sheep. All sheep in the ON inoculation experiment were negative for PrP^Sc^ by EIA of the RPLN and the brainstem at the level of the obex. A single sheep (#816, VRQ/ARQ) in the intracranial inoculation study had low positive O.D. (0.597) from the RPLN. It was later determined to be negative by IHC. Excluding two sheep that died 10 MPI, all sheep (15/15) had positive brainstem samples (range O.D. 1.739 – 4.00). All positives were confirmed by IHC.

### Lymph Nodes From TME-Infected Sheep Are Infectious

In order to rule out the possibility of undetectable infectious PrP^Sc^ within the RPLNs of sheep with TME (VRQ/VRQ) and ARQ/ARQ genotypes), we performed bioassays in ovinized (Tg338) mice. Four experimental groups of mice were inoculated with RPLN from VRQ/VRQ or ARQ/ARQ genotype sheep by either the intracranial or intraperitoneal route. Three mice intracranially inoculated with RPLN from sheep #935 (VRQ/VRQ) were positive as detected by EIA with an O.D. >4.0. None of the mice (0/18) inoculated with RPLN homogenate from sheep #808 (ARQ/ARQ) and none of the mice (0/32) intraperitoneally inoculated developed clinical signs or had detectable PrP^Sc^.

## Discussion

In the context of prion diseases, the purpose of interspecies transmission studies is to determine plausible host ranges and investigate the origins of TSE strains. This study demonstrates the susceptibility of sheep to the agent of TME by intracranial inoculation after its passage through cattle. Our findings corroborate some similarities between TME and L-BSE. These findings are congruent with previous research implicating L-BSE as the source of TME, at least the TME strain from the 1985 Stetsonville, WI outbreak (5, 17).

The tissue distribution of PrP^Sc^ in sheep with TME is similar to L-BSE in sheep. Sheep intracranially inoculated with the L-BSE agent have accumulation of PrP^Sc^ in peripheral ganglia and neuromuscular spindles; however, they lack lymphoreticular PrP^Sc^ accumulation (30, 35). Similar to sheep challenged with the L-BSE agent, all genotypes of sheep in the current study, except ARQ/ARR sheep, had immunolabeling of peripheral neuromuscular spindles and trigeminal ganglia. None of the sheep with TME had PrP^Sc^ detected in lymphoid tissue by IHC. This observation is discordant with the findings of lymphoreticular accumulation of PrP^Sc^ in sheep with C-BSE (36) and scrapie (21). One sheep (#816; ARQ/ARQ) had a low positive EIA result from a RPLN sample. Subsequent evaluation by IHC did not detect PrP^Sc^ in that tissue. However, in some TSE strains, including atypical scrapie, peripheral tissues are negative by conventional PrP^Sc^ immunodetection, but they harbor infectivity that is demonstrable by mouse bioassay (37). In this study, we examined the infectivity of RPLNs from two sheep genotypes (VRQ/VRQ and ARQ/ARQ) using a transgenic mouse bioassay. We detected infectivity in the RPLN from sheep #935 (VRQ/VRQ). None of the mice inoculated with RPLN homogenate from a sheep with the ARQ/ARQ genotype (#808) had a positive EIA result. For further characterization, subsequent passage of PrP^Sc^ from these positive bioassay mice is ongoing. We offer a possible explanation for why conventional immunoassays did not detect infectious PrP^Sc^. Perhaps immunoassays were not sensitive enough to detect small amounts of PrP^Sc^ in the tissue. A bioassay testing infectivity of RPLN from sheep #816 with the VRQ/ARQ genotype (low positive EIA result and negative IHC) is currently underway; these results will be reported separately.

The western blot migration patterns of o-bTME were identical for all sheep genotypes. We also compared o-bTME to other TSE strains that affect cattle and sheep. In both the non-enriched and acetone precipitated western blots, o-bTME resembled the inoculum, bTME. In the acetone precipitated blot, we compared multiple TSE strains of ruminants. The TME strains were similar to L-BSE, and all strains were distinct from classical scrapie in sheep.

We further scrutinized the molecular characteristics of o-bTME by staining with the N-terminus antibody, P4. Differential binding of P4 near the protease cleavage site has been used to discriminate scrapie from some BSE strains (30–33). Briefly, classical scrapie is immunoreactive with the P4 antibody while cattle derived L-BSE and C-BSE do not react. Ovine L-BSE is detectable with the P4 antibody, and therefore has a scrapie-like detectability (30). We observed that our inoculum (third pass bTME) was not detectable with the P4 antibody (C-BSE/L-like), but o-bTME samples did react with P4 (scrapie-like). The gain of N-terminal antibody binding in o-bTME but not bTME suggests that host species PrP has some influence on P4 antibody binding.

The susceptibility of sheep to the cattle passaged TME agent is similar to previous work. The transmission of the TME agent directly from mink (Idaho strain) to sheep (intracranially) was previously documented (38); however, since that experiment occurred prior to the advent of *PRNP* genotyping techniques and the widespread use of anti-PrP^Sc^ IHC detection, it lacked sheep genotype and IHC information. In the present study, all genotypes of sheep were susceptible to intracranial inoculation, and the incubation period was associated with the genotype. The duration of the incubation period by genotype (VRQ/VRQ < VRQ/ARQ < ARQ/ARQ < ARQ/ARR) was consistent with the susceptibility of sheep to classical scrapie infection (39).

We were unable to produce prion disease in sheep after oronasal inoculation with the TME agent from cattle. The possibility of oral transmission cannot be unequivocally rejected given the small sample size in our study (12 sheep). Other variables that influence susceptibility include the volume of inoculum and the age of inoculated animals (40, 41). For example, sheep older than 2–3 months old have reduced susceptibility to oral infection with BSE (42). An age-dependent decrease in susceptibility to prion disease corresponds closely with ileal Peyer's patch involution in sheep (40). The volume of the inoculum is also a factor. In this study, we used 0.1 gram (1 ml of 10% w/v solution) of inoculum that has effectively transmitted scrapie orally to sheep in previous studies (18, 23, 24). Other studies have successfully used different volumes of scrapie inoculum in sheep: 1 g repeated for 5 days (43). We cannot exclude the possibility that miniscule amounts of PrP^Sc^ are present in orally inoculated sheep because bioassays were not pursued. It's possible that after an extremely prolonged incubation period of >69 months, orally inoculated sheep could become positive, but in this study, the sheep that were sacrificed between 58 and 69 months of age lacked PrP^Sc^ immunoreactivity. There are other potential natural routes of infection that were not evaluated in the present study. For example, intralingual transmission of TME in hamsters is 100,000-times more efficient than the oral route (44). Based on that research, it is hypothesized that epithelial defects or abrasions in the tongue could predispose livestock to oral prion infection.

The bovine adapted TME agent did not appear to transmit to sheep by the oronasal route. However, concern for interspecies transmission should not be readily dismissed; the lack of transmission to one species may not accurately predict other species barriers. Case-in-point, L-BSE does not orally transmit to sheep, but L-BSE has been orally transmitted to non-human primates (45) and humanized PrP transgenic mice (46, 47). These findings indicate the potential for L-BSE zoonosis. Consequently, there should remain a concern for human and livestock exposure to the agents of L-BSE and TME, which may actually be the same agent. The susceptibility of humanized transgenic mice to o-bTME is underway and will be reported in a future manuscript.

The results of the present study indicate that sheep are susceptible to the agent of TME. Unlike classical scrapie in sheep, the agent of TME is not detectable in the lymphoid tissue using conventional immunoassay techniques. Instead non-lymphoid peripheral detection of PrP^Sc^ occurs similar to the reported distribution of experimental L-BSE in sheep. In this respect, our findings support a common origin with L-BSE. Future research utilizing a transgenic mouse model would be useful to further discriminate the observed phenotypes.

## Data Availability Statement

All datasets generated for this study are included in the article/supplementary material.

## Ethics Statement

The animal study was reviewed and approved by National Animal Disease Center Institutional Animal Care and Use Committee.

## Author Contributions

JG and JS conceived and designed the experiments. EC, JG, and JS performed the experiments. EC, JG, and SM analyzed/interpreted the data. JG contributed reagents, materials, and analysis tools. EC and JG wrote the paper. All authors edited and approved the final manuscript.

### Conflict of Interest

The authors declare that the research was conducted in the absence of any commercial or financial relationships that could be construed as a potential conflict of interest.
